# Reduced neural sensitivity to rapid individual face discrimination in autism spectrum disorder

**DOI:** 10.1016/j.nicl.2018.101613

**Published:** 2018-11-28

**Authors:** Sofie Vettori, Milena Dzhelyova, Stephanie Van der Donck, Corentin Jacques, Jean Steyaert, Bruno Rossion, Bart Boets

**Affiliations:** a Center for Developmental Psychiatry, Department of Neurosciences, KU Leuven, Leuven, Belgium; b Institute of Research in Psychological Science, Institute of Neuroscience, University of Louvain, Louvain-La-Neuve, Belgium; c Leuven Autism Research (LAuRes), KU Leuven, Leuven, Belgium; d Université de Lorraine, CNRS, CRAN, F-54000 Nancy, France; e Université de Lorraine, CHRU-Nancy, Service de Neurologie, F-5400, France

**Keywords:** Autism, EEG, Face processing

## Abstract

**Background:**

Individuals with autism spectrum disorder (ASD) are characterized by impairments in social communication and interaction. Although difficulties at processing social signals from the face in ASD have been observed and emphasized for many years, there is a lot of inconsistency across both behavioral and neural studies.

**Methods:**

We recorded scalp electroencephalography (EEG) in 23 8-to-12 year old boys with ASD and 23 matched typically developing boys using a fast periodic visual stimulation (FPVS) paradigm, providing objective (i.e., frequency-tagged), fast (i.e., few minutes) and highly sensitive measures of rapid face categorization, without requiring any explicit face processing task. We tested both the sensitivity to rapidly (i.e., at a glance) categorize faces among other objects and to individuate unfamiliar faces.

**Outcomes:**

While general neural synchronization to the visual stimulation and neural responses indexing generic face categorization were undistinguishable between children with ASD and typically developing controls, neural responses indexing individual face discrimination over the occipito-temporal cortex were substantially reduced in the individuals with ASD. This difference vanished when faces were presented upside-down, due to the lack of significant face inversion effect in ASD.

**Interpretation:**

These data provide original evidence for a selective high-level impairment in individual face discrimination in ASD in an implicit task. The objective and rapid assessment of this function opens new perspectives for ASD diagnosis in clinical settings.

## Highlights

• We assess implicit face processing in ASD via Fast Periodic Visual Stimulation EEG.
• Rapid categorization of a face as a face is not impaired in children with ASD.
• Individual face discrimination is selectively impaired in ASD.
• Children with ASD show no face inversion effect.
• FPVS-EEG opens new perspectives for clinical settings.

## 1 Introduction

The human face is a highly familiar, complex, multidimensional visual pattern, conveying a wide variety of information about an individual (identity, sex, age, mood, etc.). It constitutes arguably the most salient class of visual images for understanding perceptual categorization, a fundamental brain function. Faces can be differentiated from other objects with astounding accuracy and speed (Crouzet et al. 2010; Crouzet and Thorpe 2011; Hershler et al. 2010; Hershler and Hochstein 2005) but a more fine-grained distinction is necessary in order to differentiate among individual faces. Although there is a clear advantage at individuating familiar over unfamiliar individuals from their faces (Young and Burton 2018), neurotypical human adults are also experts at individual discrimination of unfamiliar faces (Rossion 2018). Indeed, hundreds of behavioral experiments show that, without any task training, typical human adults are highly accurate at unfamiliar face matching tasks, even in difficult tasks requiring high levels of generalization, and with similar-looking distractors (e.g., Megreya & Burton, 2006; Rossion and Michel 2018). Unfamiliar individual face discrimination is also largely affected in cases of prosopagnosia following brain damage (e.g., Sergent and Signoret, 1992b), and by simple manipulations preserving low-level visual cues such as contrast reversal (Galper 1970; Russell et al. 2006) or picture-plane inversion (Rossion 2008 for review; Yin 1969).

Given that in the human species successful social interactions require efficient decoding of information from the face, it is not surprising that deficits in face processing have been put forward as a hallmark of social difficulties in Autism Spectrum Disorder (ASD) (American Psychiatric Association 2013; Tang et al. 2015; Weigelt et al. 2012). Individuals with ASD are characterized by impairments in social communication and interaction, combined with a pattern of restricted and repetitive behavior and interests (American Psychiatric Association 2013). Many studies have tested individuals with ASD on explicit behavioral face processing tasks (e.g. see Tang et al. 2015; Weigelt et al. 2012). For instance, already in the late 1970's and early 1980's, it was observed that young children with ASD were less proficient than controls in identifying familiar peers when relying on the eye region, and that the decrease of performance for facial identity processing with inversion was smaller when compared to healty controls (Hobson et al. 1988; Langdell 1978). These impairments have generally been hypothesized to arise from a lack of interest to social stimuli such as faces early in life (Chawarska et al. 2013; Pierce et al. 2016), atypical perceptual processing strategies that favor detail processing at the cost of global holistic processing (Behrmann et al. 2006b; Behrmann et al., 2006a), and/or dysfunction of the extensive neural circuitry subtending face processing (Campatelli et al. 2013; Nomi and Uddin 2015).

However, findings from the numerous behavioral studies that have been carried out testing face processing in ASD are generally mixed and inconsistent, with some studies reporting poorer face processing abilities in ASD (Rose et al. 2007; Rosset et al. 2008; Tantam et al. 1989; Van Der Geest et al. 2002), and others reporting similar performance as neurotypical individuals (Barton et al. 2007; Falck-Ytter 2008; Guillon et al. 2014; Hedley et al. 2015; Jemel et al. 2006; Reed et al. 2007; Scherf et al. 2008; Teunisse and de Gelder 2003). Comparisons across studies are difficult due to the use of different populations (e.g. in terms of age and sex and intelligence) and the vast heterogeneity in ASD inclusion criteria, but also because of large differences in task requirements. For instance, children with ASD may be able to perform individual discrimination tasks with simultaneously presented faces, but be impaired when faces are shown consecutively (Weigelt et al. 2012). A more recent review concluded to both quantitative and qualitative differences in face recognition for individuals with ASD when compared to typically developing control participants (Tang et al. 2015). Quantitatively, the majority of reviewed studies reported reduced individual face recognition accuracy among individuals with ASD but no systematic difference in response time. Qualitatively, many studies provided evidence for the use of different face recognition strategies in individuals with ASD, as indicated by markers of atypical individual face recognition such as a reduced inversion effect (Hedley et al. 2015; Rose et al. 2007; Tavares et al. 2016; Teunisse and de Gelder 2003).

To better understand the nature of face processing impairments and to overcome the difficulty of interpreting explicit behavioral findings (which may have many sources beyond specific face processing), researchers have turned their attention for almost two decades towards implicit face processing measures such as eye-tracking (Chita-Tegmark 2016; Guillon et al. 2014), scalp electroencephalography (EEG), and functional magnetic resonance imaging (fMRI) (Campatelli et al. 2013; Nomi and Uddin 2015; Schultz 2005). Due to its relatively low cost and ease of application, EEG has been the methodology of choice for many studies in this field. While EEG studies have examined different event-related potentials (ERPs) in response to face stimuli (e.g. Benning et al. 2016; Dawson et al. 2002; Gunji et al. 2013; McCleery et al. 2009; Monteiro et al. 2017; O'Connor et al. 2007; Webb et al. 2010), the vast majority of studies focused on the N170, a negative event-related potential (ERP) peaking at about 170 ms over occipito-temporal sites following the sudden onset of a face stimulus (Bentin et al. 1996). This component is particularly interesting since it differs reliably between faces and other stimuli in neurotypical individuals (Rossion and Jacques 2011 for review) and reflects the interpretation of a stimulus as a face, beyond physical characteristics of the visual input (Caharel et al. 2013; Churches et al. 2014; Rossion 2014a). In particular, the N170 is typically right lateralized, larger in amplitude to faces as compared to non-face objects (Bentin et al. 1996; Rossion et al. 2000), and is specifically increased in amplitude and latency by picture-plane inversion of the stimulus (Rossion et al. 2000).

Unfortunately, thus far, electrophysiological studies of children or adults with ASD have failed to provide consistent evidence of abnormal N170 amplitude, latency or scalp topography in response to face stimuli (e.g. Dawson et al. 2005; Kang et al. 2018; Naumann et al. 2018; Tavares et al. 2016; Webb et al. 2010). Although a recent meta-analysis pointed to a small but significant delay in N170 latency in ASD compared to neurotypicals (Kang et al. 2018), this effect may reflect the generally slower processing of meaningful, even non-social, visual stimuli, and is quite unspecific, being found in a wide variety of psychiatric and neurological disorders regardless of diagnosis (Feuerriegel et al. 2015). Moreover, the N170 delay in response to faces may already be present in earlier visual components such as the P1, reflecting basic sensory processes (Neuhaus et al. 2016). More generally, the absolute parameters of the N170 evoked by a face stimulus (i.e., its latency, amplitude or pattern of lateralization) cannot directly index *processes* subtending social communication, such as the categorization of faces as faces, or the categorization of faces in terms of identity, emotional expression or gaze direction, etc. Thus, pertaining to the specifically underlying processes, an abnormal N170 parameter is not very informative, as it does not disambiguate the functional specificity in terms of generic face categorization, individualization or other face processes (Vettori et al. 2018). While a number of studies have shown that the N170 amplitude is sensitive (i.e., reduced) to the repetition of the same individual face (as compared to different faces) (e.g. Caharel et al. 2009; Heisz et al. 2006; Jacques et al. 2007), providing an electrophysiological index of individual face discrimination, this effect depends greatly on stimulation parameters and is not very large in typical individuals and is therefore not significant in every study, e.g.,(Amihai et al. 2011). Moreover, the N170 reduction in amplitude to repeated individual faces is difficult to identify and quantify in individual participants, and requires a relatively long recording duration to accumulate a sufficiently high number of trials.

What would be desirable at this stage to move the field forward is an implicit and yet sensitive and directly quantifiable electrophysiological measure of these specific socio-communicative face perception aspects. In the present study, we apply EEG frequency-tagging, or fast periodic visual stimulation (FPVS), to meet these requirements. The FPVS-EEG technique is based on the fairly old observation (in fact preceding standard ERP measures) that a visual stimulus presented at a fixed rate, e.g., a light flickering on/off 17 times per second (17 Hz), generates an electrical brain wave exactly at the stimulation frequency (i.e., 17 Hz in this instance), which can be recorded over the visual cortex (Adrian and Matthews 1934). The data can be transformed in the frequency domain through Fourier analysis (Regan 1966), providing highly sensitive (i.e., high signal-to-noise ratio, SNR) (Regan 1989) and objective (i.e., at a pre-determined frequency) quantifiable markers of an automatic visual process without explicit task, making it ideal to use it as a clinical diagnostic tool across age and different populations (Norcia et al. 2015; Regan, 1981, Regan, 1989; Rossion 2014a).

While this approach has long been confined to the study of low-level processes (i.e., ophthalmology and low-level vision, Norcia et al. 2015 for review) as well as their modulation by spatial and selective attention (Morgan et al. 1996; Müller et al. 2006), it has recently been extended to measure visual discrimination of more complex images, faces in particular (e.g. Rossion et al., 2012).

Besides the above-mentioned methodological advantages of the approach, the specific use of an oddball-like FPVS paradigm with complex images can provide direct measures of automatic and rapid face categorization processes with high validity and specificity. Particularly relevant for the present study is the generic face categorization paradigm (Rossion et al. 2015), yielding robust generic face categorization responses not accounted for by low-level stimulus characteristics, and the individual face discrimination paradigm, yielding robust individual face discrimination responses and a large face inversion effect in neurotypical adults (Liu-Shuang et al. 2014; Liu-Shuang et al. 2016; Xu et al. 2017).

**Fig. 1:**
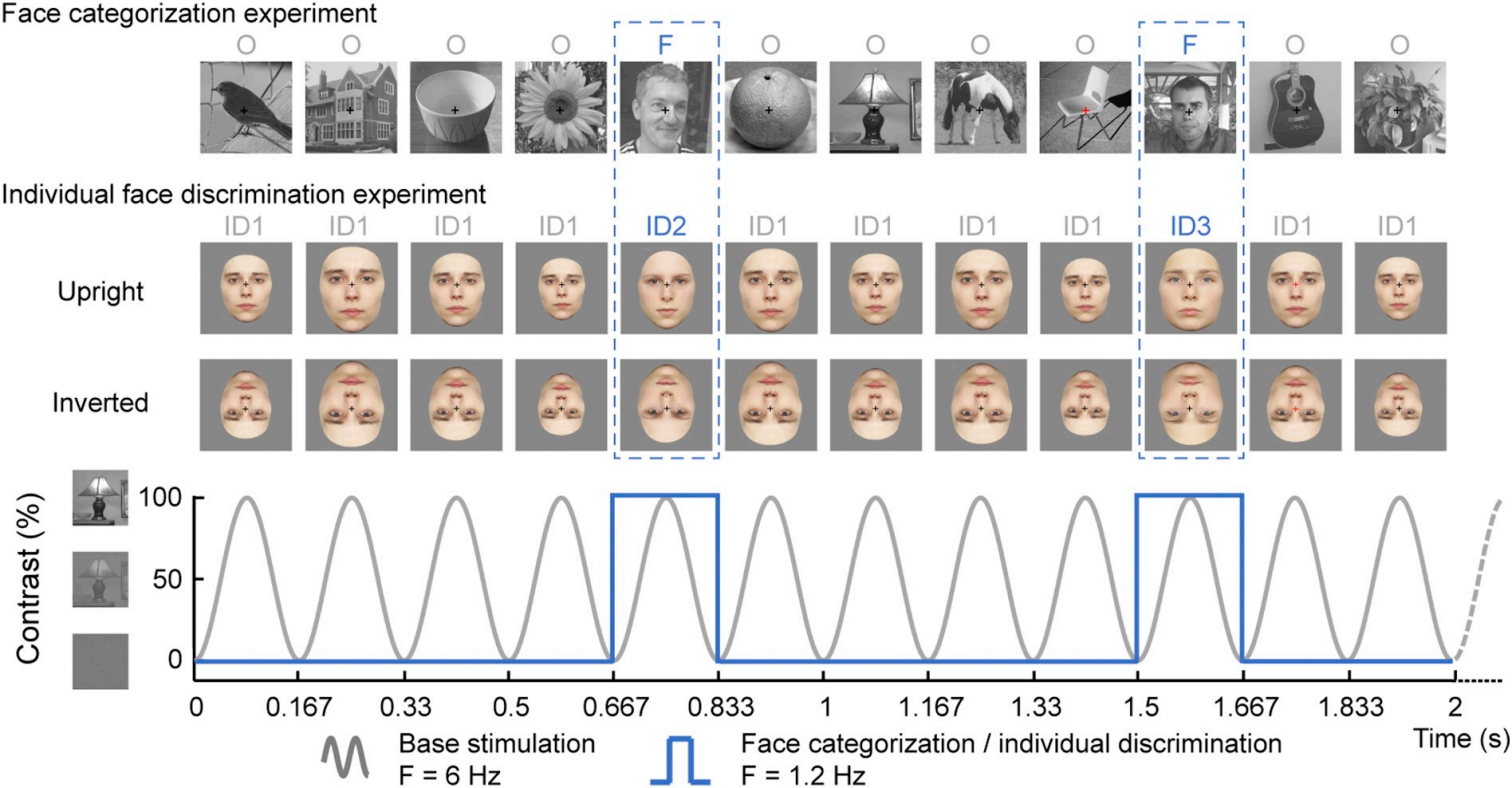
Fast periodic visual stimulation (FPVS) paradigms used in 2 separate experiments to test generic face categorization and individual face discrimination.

Capitalizing on this approach, here we tested 23 boys with ASD (8-to-12 year) and 23 matched typically developing (TD) boys with EEG recording. Each child participated in FPVS-EEG experiments assessing generic face categorization (i.e., faces vs. objects) and discrimination of unfamiliar individual faces. In both experiments, children viewed images presented one-by-one at a rate of 6 images/s (i.e. 6 Hz base rate, allowing only one fixation per face) in sequences of 40 s, while performing an orthogonal task detecting changes in the color of the fixation cross. In the generic face categorization experiment (from Rossion et al. 2015), sequences consisted of natural images of various objects, with natural face images appearing every fifth stimulus (at a rate of 6 Hz/5 = 1.2 Hz rate; Fig. 1A and movie 1 in SI). In the individual face discrimination experiment (from Liu-Shuang et al. 2014), sequences consisted of a face with a fixed identity varying in size, with faces of different identities appearing every fifth face (i.e. at 1.2 Hz, Fig. 1B and movie 2 in SI). Sequences of inverted faces provide an electrophysiological measure of the face inversion effect and allow isolating specific markers of individual face discrimination (Liu-Shuang et al. 2014).

Based on previous research with these paradigms, we expected robust general visual responses, as indicated by the 6 Hz base rate response, centered over medial occipital areas for both groups. Furthermore, we expected robust generic categorization (i.e., face-selective) and face-individuation responses over occipito-temporal electrode sites in the TD group. We also expected to observe a large inversion effect in the TD group, indicated by a decreased amplitude of the face-individuation responses for inverted faces compared with upright faces (Liu-Shuang et al. 2014). In line with those studies indicating that individual face processing may be impaired in ASD, we expected that the amplitude of the face individuation response to upright faces would be decreased in individuals with ASD and that they would show a smaller face inversion effect. Pertaining to generic face categorization, hypotheses are less specific due to a lack of studies with similar designs. On the one hand, eye-tracking studies suggests that social stimuli are processed in a less salient manner, particularly in young children with ASD (e.g. Pierce et al. 2016). On the other hand, classical EEG studies using the N170 provide a mixed pattern of basic face processing abilities in ASD, with evidence for adequate as well as abnormal processing (Kang et al. 2018). As face individualization can be dissociated from generic face categorization (as in prosopagnosia for instance; see e.g. Rossion 2014b; Rossion et al. 2011; see Liu-Shuang et al. 2016 for dissociation between the two paradigms used here), it is plausible that the level of impairment in ASD is determined by the subtlety of the underlying socio-communicative processes that are required.

## 2 Material and methods

### 2.1 Participants

We tested 46 8-to-12 year old boys, comprising 23 typically developing (TD) boys (mean age = 10.5 years ± SD = 1.2) and 23 boys with ASD (mean age = 10.6 ± 1.24, Table 1). All participants were right-handed, and had normal or corrected-to-normal vision, and had no intellectual disability. Participants with ASD were recruited through the Autism Expertise Center of the university hospital Leuven, Belgium. TD participants were recruited through elementary schools and sports clubs.

**Table 1:** Participant characteristics.

	ASD (mean ± SD)	TD (mean ± SD)	*t(df)*	*p*
Verbal IQ	103 ± 15	109 ± 12	t(44) = −1.37	0.18
Performance IQ	102 ± 16	106 ± 9	t(44) = −1.03	0.31
Total IQ	103 ± 12	107 ± 9	t(44) = −1.53	0.13
Age	10.4 ± 1.2	10.5 ± 1.2	t(44) = −0.30	0.77
Social Responsiveness Scale (*T*-score)	85 ± 12	41 ± 4	t(29.14) = −16.15	<0.0001

Participant exclusion criteria were the presence or suspicion of a psychiatric, neurological, learning or developmental disorder (other than ASD or comorbid ADHD in ASD participants) in the participant or in a first- or second-degree relative. Inclusion criteria for the ASD group were a formal diagnosis of ASD made by a multidisciplinary team in a standardized way according to DSM-IV-TR or DSM-5 criteria (American Psychiatric Association 2013) and a total *T*-score above 60 on the Social Responsiveness Scale (SRS parent version (Constantino and Gruber 2012)). Six participants with ASD took medication to reduce symptoms related to ASD and/or ADHD (Rilatine, Concerta, Aripiprazol). The TD sample comprised healthy volunteers, matched for gender, age, and verbal and performance IQ. Parents of the TD children also completed the SRS questionnaire to exclude the presence of substantial ASD symptoms. Descriptive statistics for both groups are displayed in Table 1, showing that they did not differ for age and IQ. Evidently, both groups differed highly significantly on SRS scores.

### 2.2 General procedure

The Medical Ethical Committee of the university hospital approved the study, and the participants as well as their parents provided informed consent according to the Declaration of Helsinki. All participants received a monetary reward and a small present of their choice. The session started with an assessment of intellectual abilities, followed by the two FPVS-EEG experiments and two behavioral face processing tasks. The FPVS-EEG and behavioral experiments were administered in a counter-balanced order.

### 2.3 IQ measures

An abbreviated version of the Dutch Wechsler Intelligence Scale for Children, Third Edition (WISC-III-NL; (Kort et al. 2005; Wechsler 1991)) was administered. Performance IQ was estimated by the subtests Block Design and Picture Completion, verbal IQ by the subtests Vocabulary and Similarities (Sattler 2001).

### 2.4 Behavioral measures

Two computerized behavioral face recognition tasks were administered: the Benton Facial Recognition Test (BFRT) (Benton et al. 1983) and a shortened version of the Cambridge Face Memory Test (CFMT) (Duchaine and Nakayama 2006).

The BFRT is a widely used test for face perception abilities in adults which has also been used in children (De Heering et al. 2012). We used a digitized version in which grayscale photographs were presented on a computer screen (BFRT-c (Rossion and Michel 2018)). The BFRT-c requires matching facial identities despite changes in lighting, viewpoint and size. Hence, participants cannot rely on a low-level pixel-matching strategy. Target, probe and distractor face pictures are shown simultaneously on the screen so that memory load is minimal.

In the CFMT participants also have to match faces across changes in viewpoint and illumination, but here a memory component is involved as well. To minimize the testing burden in the children, we only administered the first stage of the test. Participants are subsequently presented with three study images of the same face: frontal, left and right viewpoint, each for 3 s. Then, a display with three faces is presented, comprising one of the study images together with two other distractor faces, and participants have to select the target identity.

### 2.5 FPVS EEG experiment

Two FPVS-EEG experiments were administered in a randomized order.

#### 2.5.1 Experiment 1: generic face categorization

##### 2.5.1.1 Stimuli

The same stimuli as in Rossion et al. 2015 were used: 200 images of various non-face objects (animals, plants, man-made objects) and 50 images of faces; all within their original background. All images were centered, but differed in terms of size, viewpoint, lighting conditions and background. The entire set of stimuli is available online at http://face-categorization-lab.webnode.com/resources/natural-face-stimuli/. All stimuli were gray-scaled, resized to 200 × 200 pixels, and had equal pixel luminance and root-mean-square contrast on the whole image. Both the face and the object images were presented in a random order. At a distance of 80 cm and a resolution of 800 × 600 pixels, the stimuli subtended approximately 3.9 × 3.9 degrees of visual angle.

##### 2.5.1.2 FPVS procedure

The procedure was similar to the study of Rossion et al. (2015), except for a shorter duration of the stimulation sequences. During EEG recording, participants were seated at a distance of 80 cm from a computer monitor (24-in. LED-backlit LCD monitor). They viewed sequences of images appearing at the center of the monitor. During the sequences, stimuli were presented through sinusoidal contrast modulation at a rate of 6 Hz using in-house built software (Rossion et al. 2015). A sequence lasted 44 s, including 40 s of stimulation at full contrast flanked by 2 s of fade-in and fade-out, where contrast gradually increased or decreased, respectively. Fade-in and fade-out were used to avoid eye blinks and abrupt eye movements due to the sudden appearance or disappearance of flickering stimuli. In total, there were four sequences and the total duration of the experiment was approximately 5 min.

In each sequence, natural images of objects were presented at 6 Hz, with images of faces presented periodically as every fifth image (i.e. at 1.2 Hz = 6/5 Hz, Fig. 1, see Supplemental Movie 1). All images were drawn randomly from their respective categories, cycling through all available images before any image repeat.

The participants were instructed to fixate a black cross positioned in the center of the stimuli while continuously monitoring the flickering stimuli. They were instructed to press a key whenever they detected brief (500 ms) changes in the color of the fixation cross (which randomly occurred 10 times per sequence). This task was orthogonal to the effect/manipulation of interest and was aimed to ensure that the participants had a constant level of attention throughout the entire experiment.

#### 2.5.2 Experiment 2: individual face discrimination

##### 2.5.2.1 Stimuli

The same stimuli as in the study of Liu-Shuang et al. (2014) were used: 25 female and 25 male faces, with a neutral expression, a neutral gray background, no facial hair and cropped to remove any external features. Final images had a height of 250 pixels and a width of 186 ± 11 pixels. Shown at a distance of 80 cm, the stimuli had a visual angle of approximately 5 × 4 degrees. Inverted versions of the faces were created by vertically flipping all face images. Mean luminance of the faces was equalized online during stimulation.

##### 2.5.2.2 Procedure

Similarly to experiment 1, participants viewed sequences of images of faces presented through sinusoidal contrast modulation at a frequency rate of 6 Hz (Fig. 1, see Supplemental Video 2). In each sequence, a face of a given identity (e.g. identity A) was randomly selected and repeatedly presented. At every 5th presentation, a face of a different identity (e.g. identity B, C, D,..) was presented. Hence, changes in facial identity occurred periodically at a frequency rate of 1.2 Hz (6/5 Hz) and a sequence was as follows: AAAABAAAAC…. The experiment consisted of two conditions where faces were either presented upright or inverted (Fig. 1). Each condition was presented in four sequences (two with male faces, two with female faces). Each sequence started with a blank screen (2–5 s), then 2 s of fade-in, 40 s of full contrast stimulation and 2 s of fade-out.

The order of conditions was randomized. At each presentation cycle, the size of the face varied randomly between 80% and 120% (with 20% steps) of the original size to avoid simple image-based repetition effects and the confounding of changes in identity with changes in low-level features. Similarly to experiment 1, participants were seated at a distance of 80 cm from the computer screen, and were instructed to fixate a cross presented on the faces either between the eyes (4 sequences) or on the mouth (4 sequences). This manipulation was implemented to investigate potential group differences when fixating on the eye vs. mouth region. However, analyzing the results separately by fixation position indicated no significant effect of position, nor position by group interactions for EEG responses (see Supplemental Fig. 2). We therefore collapsed the data across both fixation positions for the main analyses.

### 2.6 EEG acquisition

EEG was recorded using a BioSemi Active-Two amplifier system with 64 Ag/AgCl electrodes. During recording, the system uses two additional electrodes for reference and ground (CMS, common mode sense, and DRL, driven right leg). Horizontal and vertical eye movements were recorded using four electrodes placed at the outer canthi of the eyes and above and below the right orbit. The EEG was sampled at 512 Hz.

### 2.7 EEG analysis

#### 2.7.1 Preprocessing

All EEG processing steps were carried out using Letswave 6 (http://nocions.webnode.com/letswave) and Matlab 2017 (The Mathworks). EEG data was segmented in 47-s segments (2 s before and 5 s after each sequence), bandpass filtered (0.1 to 100 Hz) using a fourth-order Butterworth filter, and downsampled to 256 Hz. Generally, at the group level, there were no differences in eye-blinks (*T*(44) = 0.375, *p* = 0.71). For one participant of the control group who blinked excessively (0.66 times/s, which is more than 2SD above the mean (M = 0.43 times/s, based on all participants from both groups) blinks were corrected by means of independent component analysis (ICA) using the runica algorithm (Bell and Sejnowski 1995; Makeig et al. 1996) as implemented in EEGLAB. For this participant, the first component, accounting for most of the variance, representing vertical eye movements was removed. In contrast, no participants from the ASD group blinked more than this threshold. Note that FPVS yields responses with a high SNR at specific frequency bins, while blink artefacts are broadband and thus do not generally interfere with the responses at the predefined frequency (Regan 1989). Hence, blink correction (or removal of trials with many blinks) is not performed systematically in such studies (e.g. Rossion and Boremanse 2011). Next, noisy electrodes were linearly interpolated from the 3 spatially nearest electrodes (not >5% of the electrodes, −i.e. 3 electrodes, were interpolated). All data segments were re-referenced to a common average reference.

#### 2.7.2 Frequency-domain analysis

Preprocessed data segments were further cropped to contain an integer number of 1.2 Hz cycles, beginning after fade-in until 39.1992 s (48 cycles, 10,035 time bins in total). The resulting segments were averaged for each experiment and condition separately (generic face categorization, individual face discrimination: upright, inverted), transformed into the frequency domain using a fast Fourier transform (FFT), and the amplitude spectrum was computed with a high spectral resolution of 0.025 Hz (1/40 s).

**Fig. 2:**
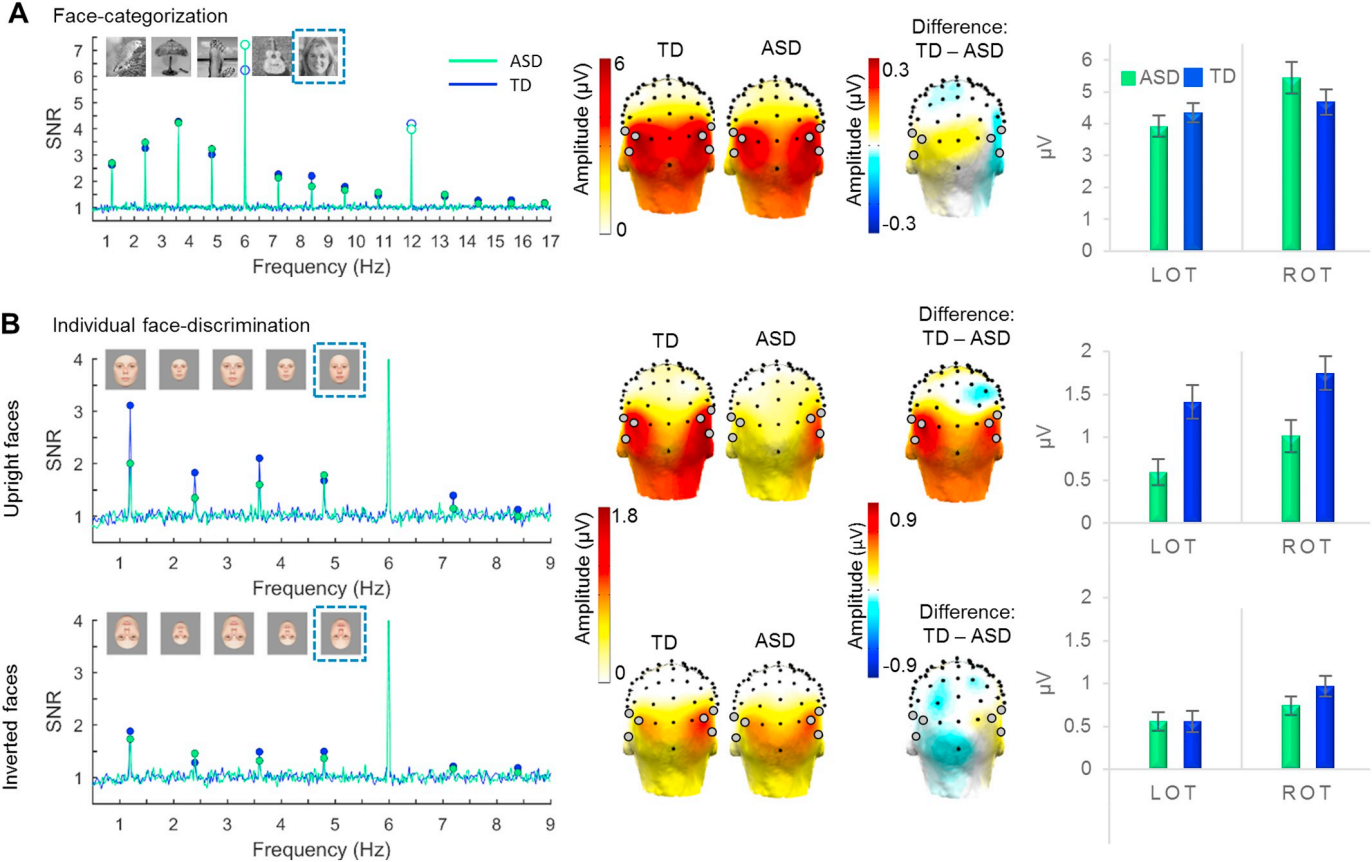
Spectral representation and scalp distribution of EEG signal during FPVS.A.Similar generic face categorization response in ASD and TD. SNR spectrum over the averaged electrodes of left and right occipito-temporal (OT) ROI (indicated with open circles on the topographical maps). ASD (green) and TD boys (blue) show similar face-selective responses, reflected by equal amplitudes at the face presentation frequency (1.2 Hz) and harmonics (2.4 Hz, 3.6 Hz, …). The response is quantified by summing the baseline-corrected amplitudes over all significant harmonics and is visualized in scalp topographies and bar graphs. Scalp topographies show that the distribution of the face-selective response is also qualitatively similar in both groups. Bar graphs (mean ± SEM) show that the amplitudes of responses in LOT and ROT are similar for both groups.B.Reduced individual face discrimination response to upright faces in ASD. SNR spectra, scalp topographies and bar graphs of left and right OT are shown for the conditions with upright and inverted faces. *: *p* < 0.05; **: *p* < 0.01.

In these experiments, the recorded EEG contains signal at frequencies that are integer multiples (harmonics) of the frequency at which images are presented (base stimulation frequency: 6 Hz) and at the frequency at which a dimension of interest is manipulated in the sequence (1.2 Hz; face appearance in experiment 1 and face identity change in experiment 2). Since the EEG response at harmonics of these frequencies reflects both the overall noise level and the signal unique to the stimulus presentation, we used 2 measures to describe the response in relation to the noise level: Signal-to-noise ratio (SNR) and baseline-corrected amplitudes (Dzhelyova et al. 2017; Liu-Shuang et al. 2014). SNR was computed at each frequency bin as the amplitude value at a given bin divided by the average amplitude of the 20 surrounding frequency bins (12 bins on each side, i.e., 24 bins, but excluding the 2 bins directly adjacent and the 2 bins with the most extreme values). Baseline-corrected amplitude was computed in the same way but subtracting the average amplitude of the 20 surrounding bins. For group visualization (Fig. 2), we computed across-subjects averages of the SNR and baseline-corrected amplitudes for each condition and electrode separately.

For amplitude quantification we first determined the range of harmonics of the 1.2 Hz and 6 Hz stimulation frequencies to consider for further analyses, based on group-level data. We determined harmonics in which the amplitude was significantly above noise using a z-score approach for each experiment separately (Dzhelyova et al. 2017; Jacques et al. 2016; Liu-Shuang et al. 2014; Liu-Shuang et al. 2016; Rossion et al. 2015): (1) FFT amplitude spectra were averaged across subjects, (2) then averaged across all electrodes and across electrodes in the relevant ROIs for each condition and experiment, and (3) the resulting FFTs were transformed in z-scores computed as the difference between the amplitude at each frequency bin and the mean amplitude of the corresponding 20 surrounding bins divided by the SD of amplitudes in these 20 surrounding bins. For each experiment separately, we quantified the response by summing the baseline-corrected amplitudes of all consecutive significant harmonics (i.e. *Z* > 1.64 or *p* < 0.05, one-tailed), see Retter and Rossion 2016).

Based on this criterion, for experiment 1 we quantified *generic face categorization* responses by summing 12 harmonics: harmonics 1 (1.2 Hz) to 14 (16.8 Hz) excluding the harmonics corresponding to the base stimulation frequency (6 and 12 Hz). For experiment 2, individual *face discrimination* responses were quantified as the sum of 6 harmonics, i.e. 1 (1.2 Hz) to 7 (8.4 Hz), excluding 6 Hz. For both experiments, the *general visual response* was quantified as the sum of the response at the base rate (6 Hz) and 2 consecutive harmonics (12 Hz and 18 Hz). Analyses performed at the individual level indicated that, despite the short recording time, in both groups every participant showed a significant (*p* < 0.01) face-categorization response. Likewise, for the identity discrimination experiment, every individual showed clear peaks at the individual face discrimination frequencies, and individual subject analyses indicated that for 41 out of 46 (22 TD, 19 ASD) participants the individual face discrimination response for faces presented at upright orientation was significant. In experiment 1, overall, the responses were higher and distributed over more harmonics than in experiment 2. This is in line with previous studies (e.g. see the study of Liu-Shuang et al. 2016, where both paradigms were also used and compared between normal observers and a prosopagnosic patient).

Based on inspection of the topographical maps of both groups (Fig. 2, Supplemental Figs. 1 and 3), and in line with previous studies using these paradigms (e.g. Dzhelyova and Rossion, 2014a, Dzhelyova and Rossion, 2014b; Liu-Shuang et al., 2014, Liu-Shuang et al., 2016; Rossion et al. 2015), EEG amplitude was quantified by regions of interest (ROI) in which the signal at multiple nearby electrodes is averaged. The analysis of the general visual response at base rate frequency (6 Hz and its harmonics) focused on three ROIs: medial occipital (MO: Oz, Iz, O1, O2), left occipital (LOT: P7, P9, P07) and right occipital (ROT: P8, P10, P08). The analysis of the generic face categorization (Exp. 1) and face individuation (Exp. 2) response at 1.2 Hz and harmonics focused on two regions of interest: LOT (P7, P9, P07) and ROT (P8, P10, P08). The electrodes in these ROIs showed the largest responses in each of the groups, suggesting the same spatial grouping (see Supplemental Fig. 3).

The baseline-corrected amplitudes in each ROI were statistically analyzed at group-level using repeated measures mixed-model ANOVAs. The general visual (base rate, 6 Hz) and generic face categorization and individual face discrimination (1.2 Hz) responses were examined separately using *ROI* (ROT, LOT, MO) and *ROI* (ROT, LOT) as within-subjects factors, respectively. For the individual face discrimination experiment, *Orientation* (upright vs. inverted faces) was an additional within-subjects factor. In both experiments, *Group* (ASD vs. TD) was a between-subjects factor for the comparison between typically developing children and children with ASD. The assumption of sphericity was checked using a Mauchly's test (with α = 0.05) and the assumption of normality of the dependent variable was checked using a standard-Wilkson test (α = 0.05). If sphericity was not met, degrees of freedom were corrected with a Greenhouse-Geisser correction. Assumptions of normality were met for all dependent variables. The assumption of homogeneity of variances was analyzed using a Levene's test (α = 0.05). For significant effects, post-hoc pairwise comparisons were conducted, using a Bonferroni correction for multiple comparisons.

In addition, we determined the significance of generic face categorization/individual face discrimination responses within the ROIs for each individual participant as follows (e.g., Dzhelyova et al. 2017): (1) the raw FFT amplitude spectrum was averaged across electrodes per ROI, and (2) cut into segments centered on the harmonics of the 1.2 Hz frequency bin surrounded by 20 neighboring bins on each side; (3) the amplitude values across 12 segments (experiment 1) and 6 segments (experiment 2) of FFT spectra were summed; (4) the summed FFT spectrum was transformed into a *z*-score using the 20 surrounding bins (see above). Response within a given ROI/participant was considered significant if the z-score at the 1.2 Hz frequency bin exceeded 2.33 (i.e., *p* < 0.01 one-tailed: signal>noise).

Finally, we applied classification models to classify individuals as belonging to the ASD or TD group. Therefore, as input variables we use the most promising outcome measures, being the amplitudes of the individual face discrimination responses. We considered three types of classification models: linear discriminant analysis (LDA), logistic regression (LR) and support vector machines (SVM), all from the scikit-learn library (Pedregosa et al. 2011). LDA is a classifier with a linear decision boundary generated by fitting class conditional probability distributions to the data. Hereby, for each class, a multivariate Gaussian probability distribution is fitted to the data, consisting of the subject-specific vectors of significant harmonics. The model classifies a subject by considering the log of the probability ratios of the class-specific probability distributions. In LR, the logs of these probability ratios are fitted by a linear model. Linear SVM is a linear classifier with the additional constraint of making the margin between the two categories as wide as possible.

## 3 Results

### 3.1 No difference in general visual base rate responses in TD and ASD

Fig. 2 displays the results for the generic face categorization and for the individual face discrimination experiments. In both experiments we observed robust brain responses at harmonics of the 6 Hz base frequency, reflecting the general response to all stimuli presented in the sequences (see Fig. 2A and B and Supplemental Fig. 1A and 1B). This response was focused on medial occipital regions, and magnitude and scalp distribution were extremely similar for both groups, yielding no significant group differences nor interactions with group (all *p* > 0.25).

### 3.2 No difference in generic face categorization responses in TD and ASD

Fig. 2A displays the results for the generic face categorization experiment, showing that the magnitude and scalp distribution of the face-selective response were virtually identical across ASD and TD groups. Responses were observed on bilateral occipito-temporal (OT) regions with maximal amplitude over electrodes PO7 (left) and PO8 (right). Repeated-measures mixed-model ANOVAs performed on averaged response amplitudes revealed no significant group differences between ASD and TD (*F*_1,44_ = 0.002, *p* = 0.96, η_p_^2^ = 0), a significant effect of Region of Interest (*ROI*) (F_1,44_ = 8.6, *p* < 0.01, η_p_^2^ = 0.16), and no *Group* by *ROI* interaction (F_1,44_ = 1.8, *p* = 0.19, η_p_^2^ = 0.04), indicating that in both groups face-selective responses were larger in right compared to left OT region.

### 3.3 Selectively reduced individual face discrimination responses in children with ASD

Fig. 2B displays the results for individual face discrimination, both for upright and inverted faces. Individual face discrimination responses were centered on bilateral OT, with a right hemisphere dominance. A repeated-measures mixed-model ANOVA with factors *Group*, *Face Orientation* and *ROI* revealed a main effect of Group (F_1,44_ = 8.45, *p* < 0.01, η_p_^2^ = 0.16), *Orientation* (F_1,44_ = 18.3, *p* < 0.001, η_p_^2^ = 0.29) and *ROI* (F_1,44_ = 14.5, *p* < 0.001, η_p_^2^ = 0.25). Crucially, the significant *Group* by *Orientation* interaction (F_1,44_ = 7.67, *p* < 0.01, η_p_^2^ = 0.15) indicated that only upright faces triggered a higher response in the TD versus ASD group (*p*_*bonferroni*_ < 0.01; Fig. 2B), whereas the response to inverted faces did not differ between groups (*p*_*bonferroni*_ = 0.55). Likewise, only the TD group displayed a significant face inversion effect with larger responses for upright compared to inverted faces (*p*_*bonferroni*_ < 0.001; ASD group: *p*_*bonferroni*_ = 0.29). There were no other significant two- or three-way interactions.

An additional ANOVA including all electrodes confirmed that there were no electrodes with a significantly larger response in the ASD group compared to the TD group. The ANOVA showed a significant interaction of *Group* by *Electrode* (*F*(63, 2772) = 2.70, *p* < 0.0001). We looked at post-hoc tests to interpret this effect. If applying a strict bonferroni correction for the number of electrodes tested (i.e., a too severe correction because the activities recorded at the different electrodes are not independent), the statistical threshold would be 0.05/64 = 0.00078. Even at this highly conservative threshold, 3 contiguous electrodes in each hemisphere show a higher response in the TD group compared to the ASD group: P7, P9, PO7, P10, PO8 and O1 (all *ps* < 0.0001).

**Fig. 3:**
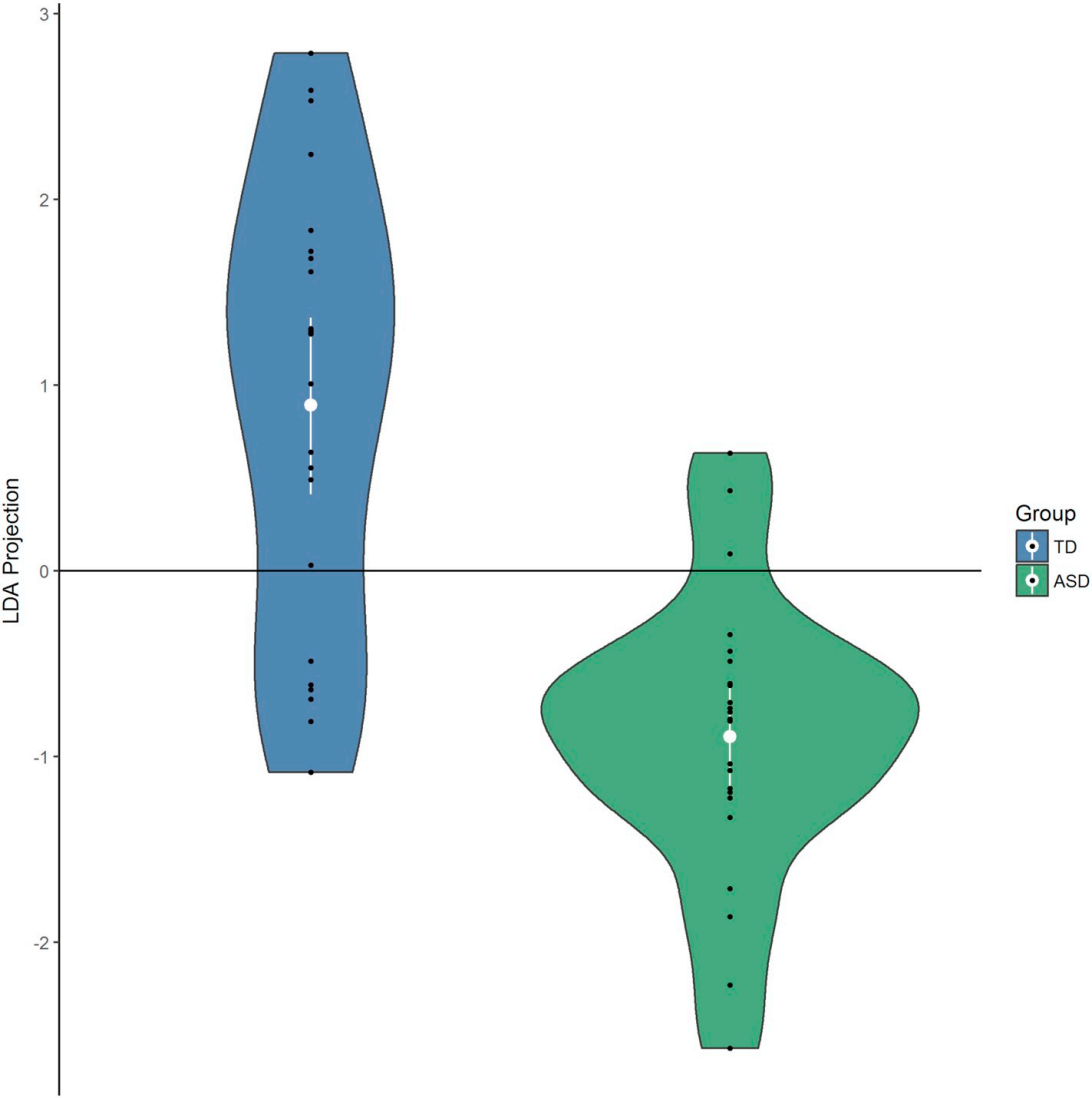
Violin plot of the ten-dimensional data of the relevant harmonics of the individual face discrimination response, projected along the LDA projection vector. The LDA was fitted to the full dataset and illustrates the separability of the groups. The horizontal line represents the decision boundary of the LDA classifier.

As the identification of a sensitive marker of impaired socio-communicative processing extends beyond statistical group differences (Kapur et al. 2012; Loth et al. 2016; McPartland 2017), we also examined how well we can predict group membership (TD vs. ASD), based on neural responses to brief identity changes in upright faces. Therefore, we used LDA, LR and SVM to classify individuals as belonging to the ASD or TD group. The ten-dimensional input vectors for the models consist of the first five harmonics of the frequency for the left and right occipito-temporal areas of interest. Harmonics are expected to be highly correlated, which is accounted for in the models. Fig. 3 demonstrates the linear separability of the ASD and TD groups, based on the LDA model. To assess the generalizability of the classification, we carried out a leave-one-out cross-validation analysis of the models, demonstrating that for LDA: 78.3%, LR: 82.6%, SVM: 87.0% of the individuals with ASD can be identified correctly (recall), and that, overall, a correct diagnostic identification of ASD vs TD (accuracy) is obtained in LDA: 73.9%, LR: 76.1%, SVM: 78.3% of the participants. Crucially, we addressed the small-sample problem and the possibility of over-fitting by performing permutation tests to statistically assess the robustness of the model (Noirhomme et al. 2014). For 10,000 permutations and two feature-selection possibilities (including the amplitude at 8.4 Hz or not), we find that the probability to find these accuracies by chance is LDA: *p* = 0.0049, LR: *p* = 0.0026, SVM: *p* = 0.0042.

We correlated the amplitude of the EEG responses to upright individual face discrimination with the scores on the SRS. While the correlation was high across groups (*r* = 0.45, *p* < 0.01), we did not find any significant correlations within the groups.

### 3.4 No group difference in control task performance and behavioral facial identity recognition

Both groups performed equally on the behavioral fixation cross change detection task, suggesting a similar level of attention throughout the experiments. Both groups showed accuracies between 91 and 96% for the two experiments with mean response times between 0.48 and 0.51 s. Statistical analyses showed no differences between the ASD group and the TD group, neither for the generic face categorization experiment (accuracy: *t*(25.79) = −0.53, *p* = 0.6; response times *t*(36) = 0.93, *p* = 0.36), nor for the face identification experiment (*t(24.45)* = −0.97, *p* = 0.34 for accuracy; *t(35)* = 1.23, *p* = 0.23 for response times).

All children also completed two behavioral face recognition tasks involving face matching and a memory component, but behavioral effects were not strong enough to reveal significant group differences (all *p* > 0.09; see Table 2).

**Table 2:** Behavioral data on explicit facial identity recognition.

	ASD (mean ± SD)	TD (mean ± SD)	*Statistic*	*p*
Cambridge face memory test accuracy				
(% correct)	0.72 ± 0.26	0.83 ± 0.17	W = 303	0.255
RT (s)	3.84 ± 1.15	4.48 ± 1.50	t(43) = 1.71	0.094
Benton facial recognition test accuracy				
(% correct)	0.71 ± 0.07	0.72 ± 0.08	t(41) = 0.60	0.552
Benton RT (s)	11.72 ± 3.22	13.53 ± 5.12	W = 270.5	0.343

*Note*. Assumptions of normal distribution of the dependent variable and homogeneity of variances were checked using a Shapiro-Wilk test and a Levene's test (both with α = 0.05) for each dependent variable separately. If the assumptions were met, behavioral data were analyzed using a *t*-test for independent samples (with α = 0.05). If the assumption of normal distribution of the dependent variable was violated a Mann-Whitney *U* test (with α = 0.05) was used. Neither the Benton face recognition test nor the Cambridge face memory test showed significant group differences in terms of accuracy and response times (RT).

## 4 Discussion

We applied FPVS-EEG to assess implicit neural face processing in children with ASD as compared to matched TD controls. Our findings reveal a dissociation between generic face categorization on the one hand and individual face discrimination on the other hand, with the ASD group being selectively impaired in the latter, more fine-grained, perceptual ability.

The base rate response across both experiments – reflecting general synchronization to the visual stimulation- was of equal amplitude for ASD and TD participants, indicating that the brains of these children similarly synchronize to the general presentation rate of visual stimuli. This response reflects a mixture of low- and high-level processes and, as in previous studies with adults, was distributed mainly over medial occipital sites, possibly due to a major contribution of early visual cortical regions (Liu-Shuang et al. 2014). The lack of difference in amplitude of this base rate response between the two groups is in line with the absence of difference in performance at the orthogonal behavioral fixation cross change detection task between the two groups, suggesting that children of both groups devoted a similar level of attention and motivation to all the tasks.

The generic face categorization response was distributed over occipito-temporal sites, slightly right lateralized, reflecting the wide distribution of face-selective neural responses across occipital and temporal cortices (Rossion et al. 2015). This response was not as clearly right lateralized as typically seen in adults (Retter and Rossion 2016; Rossion et al. 2015) and infants (de Heering and Rossion 2015), but is in line with observations in younger children (preschoolers in (Lochy et al. 2017)). The absence of amplitude difference of this generic face categorization response between the groups indicate that the brains of school-aged children with ASD are just as sensitive as those of TD children to implicitly detect socially relevant information (i.e. faces) among a stream of non-social images. These results fit with evidence from other implicit social paradigms, such as the social preference eye-tracking studies, showing that social orienting is not qualitatively impaired in school-aged children with ASD (Guillon et al. 2014).

For both groups, the individual face discrimination response (signaling the neural sensitivity for differences in facial identity) was distributed over occipito-temporal cortices and was clearly right lateralized, in agreement with the well-established right hemispheric dominance of face perception in humans (e.g. Bentin et al. 1996; Jonas et al. 2016; Meadows 1974; Sergent & Signoret, 1992). By presenting facial images at 6 Hz (~ 167 ms per face), the idiosyncratic characteristics of novel faces need to be grasped at a single glance. For upright faces, this rapid and automatic individual face discrimination is typically facilitated by the perception of the face as a whole, or as a single representation undecomposed in features (“holistic face perception” (Rossion 2013 for review; Sergent 1984; Tanaka and Farah 1993; Young et al. 1987). When faces are presented upside down, however, holistic face perception is impaired, despite preserving the low-level properties of the images. The ASD group showed reduced facial identity discrimination responses only for the upright faces and not for the inverted faces. Accordingly, in boys with ASD, much like in brain-damaged patients with prosopagnosia (Busigny and Rossion 2010), rapid discrimination of upright faces is not superior to inverted faces, suggesting that individuals with ASD may employ atypical processing strategies when individuating faces (Evers et al. 2018; Tang et al. 2015).

Note that this group difference cannot be explained by different levels of attention or motivation, or by general differences in neural synchronization to visual stimulation. Likewise, the selective deficit in upright facial identity discrimination cannot be explained by an inability to reliably detect discrimination responses in the ASD group, since the two groups did not differ in the generic categorization of faces and in the discrimination of inverted faces. These findings highlight the importance of studying face processing by a broader series of tasks, as the exclusive use of the generic face categorization paradigm would have led to the wrong conclusion that boys with ASD present intact face processing.

This study illustrates the strength of the FPVS-EEG approach as compared for instance to a standard EEG approach with slow non-periodic stimuli leading to components (ERPs) analyzed in the time-domain (Regan 1989; Retter and Rossion 2016; Rossion 2014a). With FPVS-EEG, the process of interest is identified objectively because the frequency is known in advance by the experimenter. Moreover, the response can be quantified directly in the frequency domain without having to define time-windows based on participants' specific responses. The technique is also highly sensitive because a large number of discriminations can be presented in a very short amount of time. Most importantly, with a high frequency resolution, the response of interest falls into a tiny frequency bin (and harmonics) containing very little noise, since the noise is distributed into numerous frequency bins (Regan 1989). This high sensitivity of the approach allows to obtain significant responses found in virtually every single participant following a few minutes of testing (Liu-Shuang et al. 2014; Xu et al. 2017). The response is also highly specific, dissociating general visual activity (at the base rate) from responses reflecting generic face categorization (Rossion et al. 2015), individual face discrimination (Liu-Shuang et al. 2014), or else facial expression discrimination (Dzhelyova et al. 2017), without the need to subtract a control condition from the condition of interest. Finally, the paradigms measure these functions under severe time constraints, i.e. here a single glance at a face, and without explicit task, mimicking the speed and automaticity of these processes in real life without adding unreasonable pressure for behavioral responses in the population tested.

Our results indicate that the FPVS EEG approach is able to rapidly pinpoint face processing impairments in ASD, which may be invisible in explicit behavioral recognition tasks. Here, crucially, the impairments in ASD were confined to more subtle socio-communicative cues, such as the (holistic) neural processing of facial identity. One might question why these specific effects are not reflected in the behavioral face recognition tasks in our sample. This may be due to the explicit nature of these behavioral tasks, allowing compensatory strategies and the influence of other factors beyond face processing, such as motivation and attention (Weigelt et al. 2012). This is also illustrated by the weak correlation between both behavioral face processing measures. Previous behavioral research has shown that explicit tasks are often not discriminative between TD and ASD groups (Weigelt et al. 2012). Hence, implicit tasks might better reflect how faces are processed on a daily life basis. Against this background, a recent study investigated the association between the FPVS-EEG face individuation response and performance on the CFMT, and concluded that both tasks do share some common variance but are not strongly related. In particular, the FPVS response captures the perceptual processes involved in facial identity discrimination, while the CFMT is an explicit and cognitively complex memory task requiring memory and decision-making processes that go beyond the mere perceptual differentiation of face identity (Xu et al. 2017). In addition, due to time constraints, the children in our study only completed the first part of the CFMT, which is typically the easiest part, characterized by the highest performance (Bowles et al., 2009), thus possibly less sensitive to observe clear group differences.

While the correlation between EEG face identity discrimination responses and SRS scores was significant across the groups, we did not find any significant correlations within the groups. We believe that this is due to two factors. First, the limited variation in SRS scores within each of the groups. This is partly due to our inclusion criterion being a cut-off for each group: boys in the ASD group had a total SRS T-score higher than 60, while TD boys all had a score lower than 60. Moreover, the SRS measures the severity of ASD symptoms over a variety of domains, based on evaluations by the parents. Hence, while it gives a clear idea of the perceived symptoms in daily life, this measure does not purely reflect the actual behavior and performance, and is also determined by several other parent-related processes (e.g. whether there are other children in the family with an ASD diagnosis) (De la Marche et al. 2015). Second, although the EEG individual face discrimination response reflects a highly selective automatic process, the variations of amplitude of this response across individuals also reflects general factors such as skull thickness and cortical folding (see the discussion in Xu et al. 2017). While these factors should be neutralized when comparing relatively large groups of participants (or comparing different paradigms in the same participants), they add variance to amplitude differences within a group of individuals, reducing the significance of correlation measures.

The use of a well-selected, well-matched and homogeneous participant sample in terms of age, gender, IQ, and diagnostic status is certainly an asset. It allowed us to observe clear differences in the neural individual face discrimination response. In comparison, a recent study with a similar FPVS-EEG approach (where only individual face discrimination was tested) failed to find such differences in adults with ASD (Dwyer et al. 2018). Yet, in that study, participants were both male and female adults with a variable age range. Moreover, the patient group comprised self-selected individuals who themselves reported having a diagnosis of ASD, but without any formal professional multidisciplinary assessment. In contrast, in our study, all patients in the ASD group had a formal and recently confirmed diagnosis of ASD, as assessed by the multidisciplinary team of the University Hospitals.

Against this background, one may question whether the findings will generalize to the broader autism population. Further studies will of course be required in order to address this issue. Importantly, however, the advantages of the FPVS approach offer a unique opportunity to obtain data in low-functioning individuals with ASD, as well as in young children and infants (de Heering and Rossion 2015; Lochy et al. 2017). Furthermore, in the longer term, the discrimination responses obtained with FPVS-EEG yield the potential to be used as a biomarker, possibly for the early detection of ASD. Indeed, when the individual data were taken into account, the classification analyses missed only a few participants with ASD, thus showing a great potential for individual classification. Evidently, for this purpose, the sensitivity and specificity of the approach should further be improved, possibly by incorporating data of additional FPVS-EEG paradigms that also show discriminative value.

## 5 Conclusions

While showing typical generic face categorization responses, individuals with ASD were impaired at rapid individuation of faces, a crucial aspect of daily life social interactions. Given the strength of the effects obtained, the implicit nature of the measure and the straightforward application and analysis, the presented FPVS-EEG approach opens an avenue for studying populations that are not susceptible to explicit verbal instructions and hence less accessible for research, such as infants and people with low-functioning ASD.

## Declaration of interest

The authors declare no competing interests.

## Appendix A Supplementary data

Supplementary material Image 1
