# Supplementary material for: Reduced neural sensitivity to rapid individual face discrimination in autism spectrum disorder
Source: Neuroimage Clin. 2018 Nov 28;21:101613. doi: 10.1016/j.nicl.2018.101613 (PMC6411619; doi:10.1016/j.nicl.2018.101613)
Supplement: Supplementary file 1 — Supplementary material [file mmc1.docx]

**SUPPLEMENTAL INFORMATION**

**Fig. S1. Spectral representation and scalp distribution of EEG base rate signal during FPVS.**

**
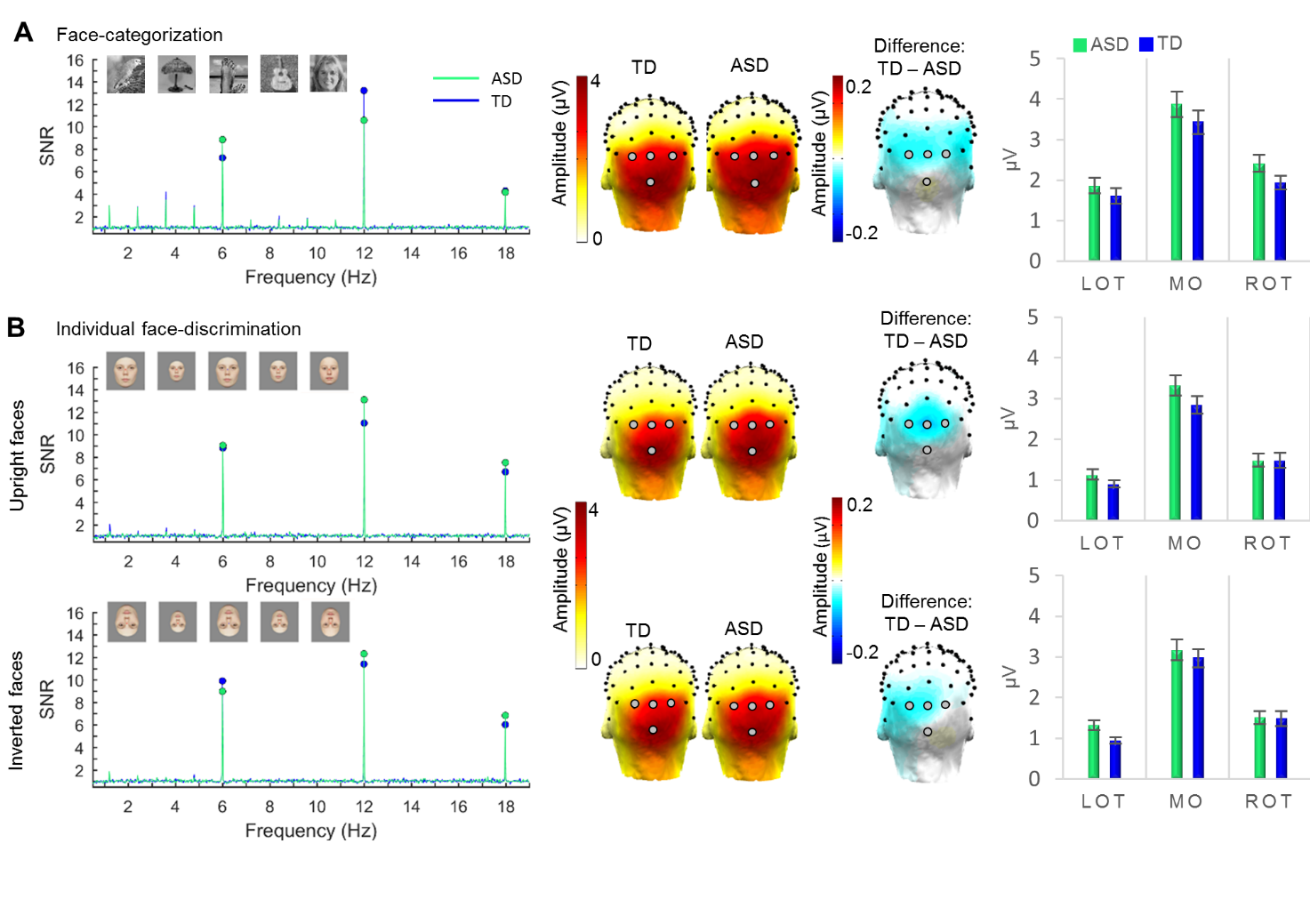
**

In addition to oddball signals at 1.2 Hz and harmonics, we also observed a high SNR response at harmonics of the 6 Hz base stimulation frequency, reflecting the general visual response to all stimuli presented in the sequences. This response was focused on the medial occipital (MO) region for both populations, hence an MO ROI was defined (indicated with open circles on the topographical maps). Magnitude and scalp distribution of the general visual response were extremely similar between ASD and TD groups. All p-values are corrected for multiple comparisons using the bonferroni correction (*p_b_*).

1. Generic face categorization experiment: SNR spectra, scalp topographies and bar graphs of the averaged base rate amplitudes for three ROIs (left occipito-temporal, LOT; medial occipital, MO; and right occipito-temporal, ROT). A repeated-measures mixed-model ANOVA on averaged response amplitudes revealed a significant effect of ROI (*F_1.7,76.7_ =* 76.15*, p_b_ <* .001*, η_p_^2^* =.63) but no effect of group (*F_1,44_* = 1.996*, p_b_* = .165*, η_p_^2^* =.045), nor group by ROI interaction *(F_2,76.7_* = .257*, p_b_* = .746*, η_p_^2^* = .006*)*, indicating that in both groups, the response is largest over MO compared to LOT (*p*_b_ < .001) and ROT (*p*_b_ < .001).
2. Individual face discrimination: SNR spectra, scalp topographies and bar graphs of the averaged base rate amplitudes for the three ROIs. A repeated-measures mixed-model ANOVA with factors group, face orientation and ROI showed no significant effect for group (*F_1,44_* = 1.29, *p_b_* = 0.26, *η_p_^2^* = 0.03) nor orientation (*F_1,44_* = 2.453, *p_b_* = 0.125, *η_p_^2^* = 0.055), and no significant interaction involving *group* or *orientation:*  *ROI* x *orientation* (*F*_2,88_ = 1.1; *p_b_* = .34; η_p_^2^ = .024); *ROI* x *group* (*F*_2,88_ = .9; *p_b_* = .41; η_p_^2^ = .02) and *orientation* x *group* (*F*_1,44_ = .15; *p_b_* = .705; η_p_^2^ = .02). There only was a significant main effect for *ROI* (*F*_2_*_,88_* = 125.6; *p_b_* < .001; *η_p_^2^* = .74), with general visual responses being highest in the MO region (*p_b_* < 0.001 for comparisons to ROT and LOT), followed by ROT (vs. LOT: *p_b_* < 0.01) and then LOT.

**Fig. S2. Averaged oddball amplitudes for individual face discrimination in relation to the positioning of the fixation cross.**
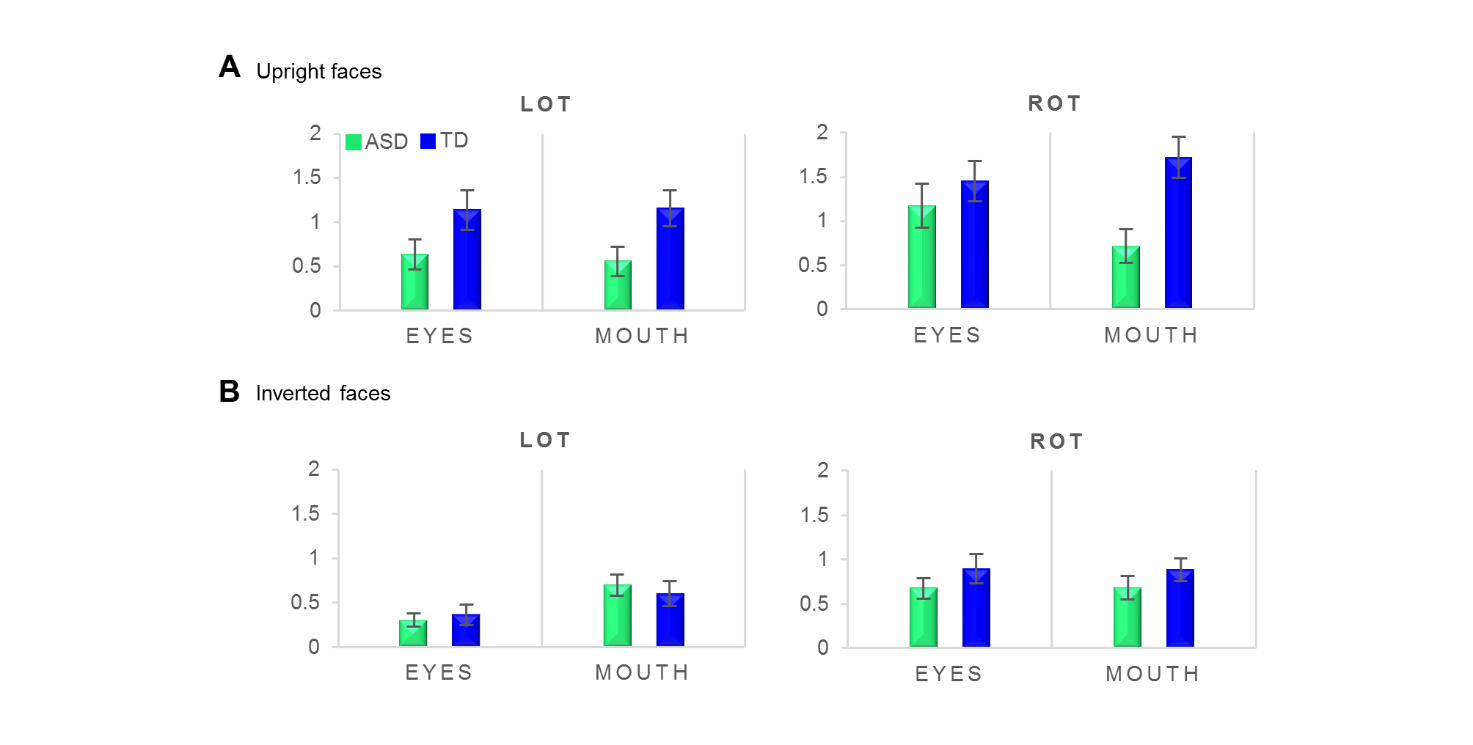


In the individual face discrimination experiment, we varied the position of the fixation cross. Within each condition, the cross was positioned just below the eyes (i.e., optimal fixation for generic face categorization tasks, see Peterson & Eckstein, 2012) in four of the trials, and on the mouth in the other four trials. A repeated-measures mixed-model ANOVA showed no main effect of the position of the fixation cross (*F_1,42_* = .233, η_p_^2^ = .006, *p_b_* = .632), nor any interactions of this factor with *Group* (*F_1,42_* = 1.376, η_p_^2^ = .032, *p_b_* = .247) or *ROI (F_1,42_* = .405, η_p_^2^ = .01, *p_b_* = .528) or *Orientation* (*F_1,42_* = 2.346, η_p_^2^ = .053, *p_b_* = .133). Also when fixation position was included in the model as a factor, the overall effect of *Group* (*F_1,42_* = 5.301, η_p_^2^ = .112, *p_b_* = .026) and the critical interaction effect of *Group* x *Orientation* (*F_1,42_* = 6.507, η_p_^2^ = .134, *p_b_* = .014) were observed.

**Fig. S3. 2D topographical maps of the face identification response for the ASD and the TD group.**

Each map is shown with its respective scale where the upper limit represents the maximal amplitude. The electrodes associated with the largest responses were exactly the same in both populations, hence suggesting that both groups have the same spatial grouping. The ROIs were based on these maximal electrodes for both groups. Moreover, this is in complete agreement with previous scalp topographies obtained in this paradigm in previous studies in adults (Liu-Shuang et al., 2014; Liu-Shuang et al., 2016; Dzhelyova & Rossion, 2014a; Dzhelyova & Rossion, 2014b).


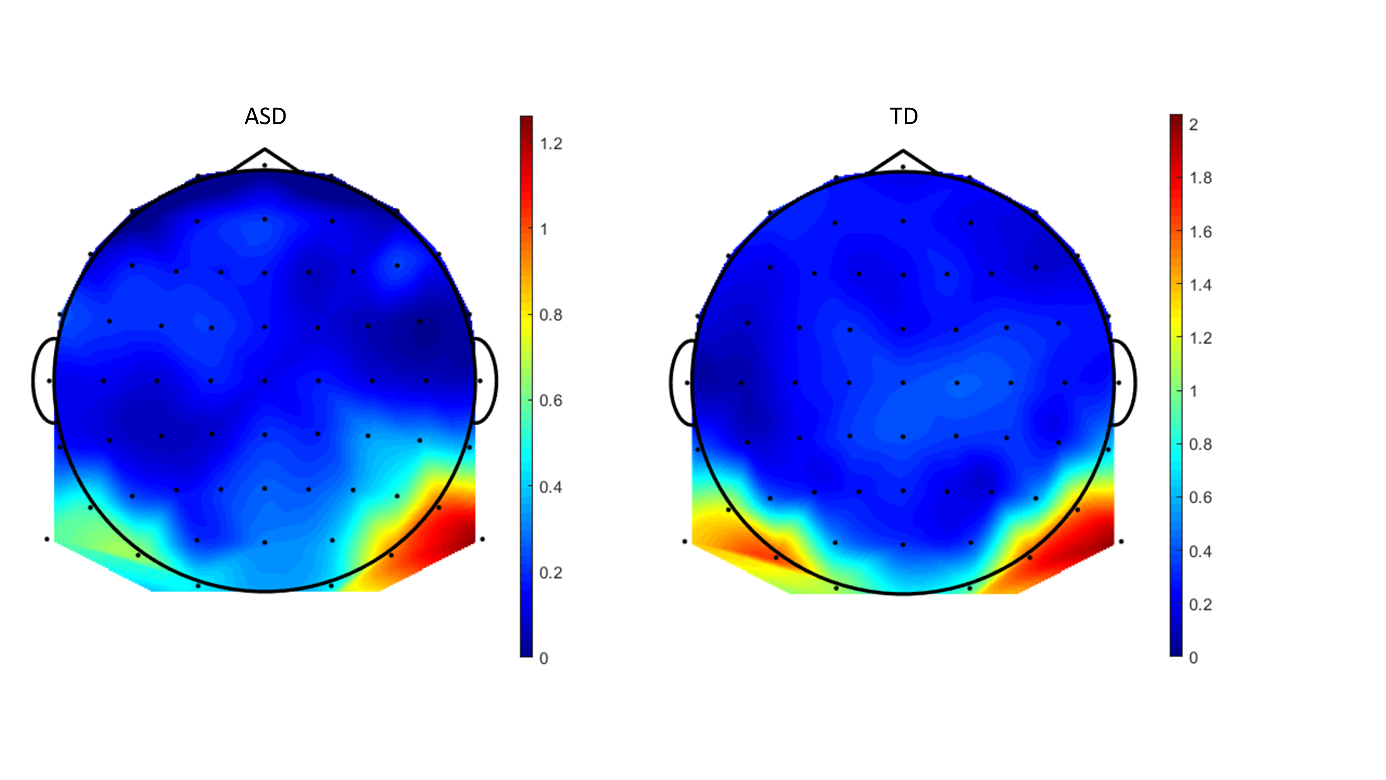


Movie S1. Generic face categorization

SI_Mov1

Movie S1. Face identity discrimination

SI_Mov2
